# Teenage exercise is associated with earlier symptom onset in dysferlinopathy: a retrospective cohort study

**DOI:** 10.1136/jnnp-2017-317329

**Published:** 2018-01-29

**Authors:** Ursula R Moore, Marni Jacobs, Roberto Fernandez-Torron, Jiji Jang, Meredith K James, Anna Mayhew, Laura Rufibach, Plavi Mittal, Michelle Eagle, Avital Cnaan, Pierre G Carlier, Andrew Blamire, Heather Hilsden, Hanns Lochmüller, Ulrike Grieben, Simone Spuler, Carolina Tesi Rocha, John W Day, Kristi J Jones, Diana X Bharucha-Goebel, Emmanuelle Salort-Campana, Matthew Harms, Alan Pestronk, Sabine Krause, Olivia Schreiber-Katz, Maggie C Walter, Carmen Paradas, Jean-Yves Hogrel, Tanya Stojkovic, Shin’ichi Takeda, Madoka Mori-Yoshimura, Elena Bravver, Susan Sparks, Jordi Diaz-Manera, Luca Bello, Claudio Semplicini, Elena Pegoraro, Jerry R Mendell, Kate Bushby, Volker Straub

**Affiliations:** 1 The John Walton Muscular Dystrophy Research Centre, MRC Centre for Neuromuscular Diseases, Institute of Genetic Medicine, Newcastle upon Tyne, UK; 2 Children’s Research Institute, Division of Biostatistics and Study Methodology, Children’s National Health System, Washington, District of Columbia, USA; 3 Department of Pediatrics, Epidemiology and Biostatistics, George Washington University, Washington, District of Columbia, USA; 4 Neuromuscular Area, Biodonostia Health Research Institute, Neurology Service, Donostia University Hospital, Donostia-San Sebastian, Spain; 5 The Jain Foundation, Seattle, Washington, USA; 6 Children’s Research Institute, Division of Biostatistics and Study Methodology, Children’s National Health System, Washington, District of Columbia, USA; 7 AIM and CEA NMR Laboratory, Institute of Myology, Pitié-Salpêtrière University Hospital, Paris, France; 8 Magnetic Resonance Centre, Institute for Cellular Medicine, Newcastle University, Newcastle Upon Tyne, UK; 9 Charite Muscle Research Unit, Experimental and Clinical Research Center, The Max Delbrück Center for Molecular Medicine, Berlin, Germany; 10 Department of Neurology and Neurological Sciences, School of Medicine, Stanford University, Stanford, California, USA; 11 Institute for Neuroscience and Muscle Research, Children’s Hospital at Westmead, University of Sydney, Sydney, New South Wales, Australia; 12 Department of Neurology, Children’s National Health System, Washington, District of Columbia, USA; 13 National Institutes of Health (NINDS), Bethesda, Maryland, USA; 14 Department of Neuromuscular and ALS Center, La Timone Hospital, Aix-Marseille Université, Marseille, France; 15 Department of Neurology, Washington University School of Medicine, St. Louis, Missouri, USA; 16 Department of Neurology, Friedrich-Baur-Institute, Ludwig-Maximilians-University of Munich, Munich, Germany; 17 Neuromuscular Unit, Hospital Universitario Virgen del Rocío/Instituto de Biomedicina de Sevilla, Sevilla, Spain; 18 Institut de Myologie, AP-HP, Groupe Hospitalier Pitié-Salpêtrière, Boulevard de l’Hôpital, Paris, France; 19 Department of Neurology, National Center Hospital, National Center of Neurology and Psychiatry, Tokyo, Japan; 20 Carolinas Healthcare System Neurosciences Institute, Charlotte, North Carolina, USA; 21 Centro de Investigación Biomédica en Red en Enfermedades Raras (CIBERER), Barcelona, Spain; 22 Neuromuscular Disorders Unit, Neurology Department, Hospital de la Santa Creu i Sant Pau, Barcelona, Spain; 23 Department of Neuroscience, University of Padova, Padova, Italy; 24 Nationwide Children’s Hospital, Columbus, Ohio, USA

**Keywords:** neuromuscular, muscular dystrophy

## Introduction

Dysferlinopathy, an autosomal recessive muscular dystrophy caused by *DYSF* mutations, demonstrates a variable phenotype and progression rate, with symptom onset ranging from first to eighth decade and some patients requiring wheelchairs for mobility within 10 years, with others remaining minimally affected.^1^

Dysferlinopathy populations have previously been described as having an unusually high level of presymptomatic sporting ability.^2^ We hypothesised that this activity could be related to subsequent disease progression and investigated the hypothesis using data from the Jain Foundation’s Clinical Outcomes Study (COS) of 202 patients with dysferlinopathy.^1^

## Methods

Data were used from 182 of the 202 patients enrolled in the Jain COS; 10 dropped out and did not give permission to use their data and 10 did not fully complete the exercise questionnaire.

The questionnaire used in the screening visits (online supplementary information) between 6 November 2012 and 19 March 2015 asked about the type, level and frequency of all physical activity prior to symptom onset. Self-reported age of first symptoms, first wheelchair use and full-time wheelchair use was taken from screening questionnaires.

Exercises were classified based on metabolic equivalents (METs) as moderate (MET 3–6) or vigorous (MET >6) (online supplementary table 1).^3^ Participants were coded, based on the maximum frequency of activity reported between ages 10 and 18 years, as 0—no physical activity; 1—vigorous activity occasionally/monthly, or moderate activity once weekly; 2— moderate activity multiple times per week or vigorous activity once weekly; and 3—vigorous activity multiple times per week.

10.1136/jnnp-2017-317329.supp1 Supplementary file 1

### Statistical analysis

Age of symptom onset was compared by analysis of variance (ANOVA) with least squares means for individual group differences. Risk of symptom onset, occasional wheelchair use and full-time wheelchair requirement over time were compared for exercise groups 1, 2 and 3 against group 0 using Cox proportional hazards regression. Proportional hazards assumption was violated for initial wheelchair use. Inspection of survival curves suggested this was occurring at later ages; thus, analysis was rerun censoring at age 50 if there was no wheelchair use up to that point, which prevented violation of this assumption while capturing 46/55 events.

Interaction between teen exercise level, gender and clinical diagnosis was also assessed by two-way ANOVA. Subgroups of limb girdle muscular dystrophy 2B (LGMD2B), Miyoshi myopathy (MM) or ‘other’ (all genetically confirmed dysferlinopathies) were used for analysis.

## Results

Exercise group 0 had more female patients (65%). Demographic characteristics of each exercise group were otherwise similar (online supplementary table 2).

10.1136/jnnp-2017-317329.supp2 Supplementary file 2

### Age of symptom onset

**Figure 1:**
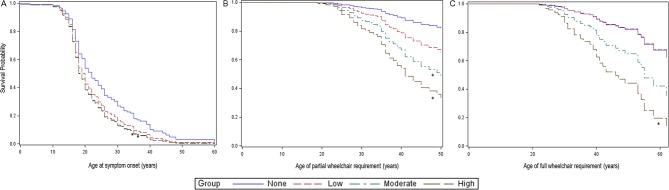
Risk of symptomatic disease and wheelchair use over time in dysferlinopathy. Graphs show the probability of remaining event free during the time under study using Cox proportional hazards regression. Events are the onset of symptoms (A), the first time a wheelchair is used (B) and the need to use a wheelchair full time (C). Survival probabilities significantly different from exercise group 0 are marked with an asterisk (*). For first-time wheelchair use (B), this graph shows the results excluding those patients over 50 years of age at the screening visit as including these patients led to a violation of the proportional hazards assumption.

Estimated mean age of symptom onset differed by group (P=0.03) and was later in group 0 (mean 24.8 (95% CI 22.3 to 27.2)) compared with groups 2 (20.2 (18.1 to 22.3), P=0.006) and 3 (20.6 (18.4 to 22.8), P=0.01), but not 1 (21.7 (17.7 to 25.7), P=0.20). Cox regression analysis suggested that groups 2 (HR 1.56 (95% CI 1.06 to 2.30)) and 3 (HR 1.54 (1.04 to 2.30)) were at increased risk of earlier symptom onset than group 0 (figure 1). This was not significant for group 1 (HR 1.38 (0.78 to 2.45)).

In patients with a clinical diagnosis of LGMD2B, groups 1–3 all showed a significantly increased risk of earlier onset compared with group 0 (1: HR 7.74 (95% CI 3.07 to 19.49); 2: HR 1.71 (1.05 to 2.77); 3: HR 1.91 (1.14 to 3.18)). Significant associations were not seen among those with a diagnosis of MM or ‘other’.

Results were independent of gender, which was not significantly related to age of onset (P=0.329) or exercise group (P=0.328).

### Wheelchair use

Fifty-five patients reported age at first wheelchair use and 42 using a wheelchair full time.

Statistical analysis showed a higher risk for part-time wheelchair use in exercise groups 2 (HR 3.57 (95% CI 1.36 to 9.36)) and 3 (HR 5.44 (2.20 to 13.48)) compared with group 0; no association was noted for group 1 (HR 2.12 (0.53 to 8.51)).

Greater teenage activity was also associated with increased risk of full-time wheelchair requirement in group 3 (HR 4.11 (1.75 to 9.64)). This was not significant in groups 1 (HR 0.97 (0.20 to 4.73)) or 2 (HR 2.18 (0.82 to 5.75)).

## Discussion

In the present study, patients recalling greater teenage exercise levels demonstrated increased risk for earlier symptom onset and wheelchair requirement. Teenage exercise level was chosen as this is typically before symptom onset in dysferlinopathy, yet old enough for significant exercise regimens to have started. Although an association between exercise and subsequent disease progression has previously been suggested by Angelini *et al* 4 Klinge *et al* 2 reported no significant effect of exercise on age of symptom onset in 36, mostly LGMD2B, patients. These studies all rely on patient recall, introducing potential question and recall bias. We tried to mitigate for this using a scoring system based on METs rather than Klinge’s use of patient-reported sporting level. However, our approach still does not produce uniform exercise categories, requiring grouping of different types and frequencies of exercise, which may not have equitable physiological impact. Unfortunately, this is the nature of a retrospective study, which is necessary while so few patients are diagnosed presymptomatically.

Exercise is usually considered beneficial in muscular dystrophies, and so this finding in dysferlinopathy may suggest a unique underlying pathological mechanism. Investigation of this was not within the scope of this study. However, previous work has suggested that dysferlinopathy may increase aptitude for training early in life, but the more a patient exercises vigorously, the more muscle damage occurs, which is then inadequately repaired^5^—accelerating both disease onset and symptom progression.

This study raises implications for patients and families. If intensive exercise causes earlier onset and faster progression, asymptomatic patients should consider limiting their exercise and affected siblings should be identified to allow for early disease-modifying advice. However, as the HRs are small, this needs to be balanced against the loss of other lifestyle benefits of exercise. As we did not look at the effects of exercise once symptoms began, we would not advocate that symptomatic patients stop exercising.

This letter describes an association of intensive exercise during the teenage years with earlier disease onset and faster rate of disease progression in patients with dysferlinopathy.
