# Supplementary material for: Teenage exercise is associated with earlier symptom onset in dysferlinopathy: a retrospective cohort study
Source: J Neurol Neurosurg Psychiatry. 2018 Jan 29;89(11):1224–6. doi: 10.1136/jnnp-2017-317329 (PMC6227796; doi:10.1136/jnnp-2017-317329)
Supplement: Supplementary file 1 [file jnnp-2017-317329supp001.pdf]

Supplementary table 1 – reported exercises and there classification

Activity	performance*	MET category (1 = Moderate, 2 = Vigorous)
American football	Any	2
Athletics (track and field)	Any	2
Badminton	Any	1
Ballet	Any	1
Baseball	Any	2
Basketball	Average or worse	1
Basketball	Better than average	2
Cricket	Any	1
Cycling	Average or worse	1
Cycling	Better than average	2
Dancing	Average or worse	1
Dancing	Better than average	2
Diving	Any	1
Figure skating	Average or worse	1
Figure skating	Better than average	2
Golf	Any	1
Gymnastics	Any	1
Handball	Any	2
Hockey	Any	2
Horseback riding	Average or worse	1
Horseback riding	Better than average	2
Martial arts	Any	2
Rowing	Average or worse	1
Rowing	Better than average	2
Rugby	Any	2
Running	Any	2
Soccer/football	Any	1
Step aerobics	Any	2
Swimming	Average or worse	1
Swimming	Better than average	2
Table tennis	Any	1
Tennis	Average or worse	1
Tennis	Better than average	2
Volleyball	Any	1
Skiing	Average or worse	1
Skiing	Better than average	2
Weight lifting	Average or worse	1
Weight lifting	Better than average	2
Yoga/pilates	Any	1
Boxing/wrestling	Any	2
Outdoor activities	Average or worse	1
Outdoor activities	Better than average	2
"Water aerobics"	Any	1
"Lacrosse"	Any	2
"Marching band"	Any	1

Table 1: Sports reported by patients and each sports metabolic equivalent score. Moderate activities (MET score 1 in table) have a metabolic equivalent of 3-6 (3.5-7kcal/min) and vigorous activities (MET score 2 in a table) >6 METs (>7kcal/min). Some activities can be either moderate or vigorous depending on

intensity at which they are performed. For these activities, level of performance was used as an indicator of intensity with average or less than average performance likely to suggest sport done at lower intensity than above average, regional or national representation at a sport.
