# Supplementary material for: Teenage exercise is associated with earlier symptom onset in dysferlinopathy: a retrospective cohort study
Source: J Neurol Neurosurg Psychiatry. 2018 Jan 29;89(11):1224–6. doi: 10.1136/jnnp-2017-317329 (PMC6227796; doi:10.1136/jnnp-2017-317329)
Supplement: Supplementary file 2 [file jnnp-2017-317329supp002.pdf]

Supplementary Table 2 – group demographics at baseline visit										
**Exercise group**	**Total number of patients**	**Mean age (SD)**	**% male**	**Number using wheelchair occasionally (%)**	**Number using wheelchair full time (%)**	**'non-ambulant' (%)**	**Number with symptom onset <15 yrs**	**Clinical diagnosis**		
								**LGMD2b**	**MM**	**other**
**0**	46	43 (14)	35	10 (22%)	7 (15%)	8 (17%)	6 (13%)	29 (63%)	14 (30%)	3 (7%)
**1**	17	38 (15)	53	5 (29%)	2 (12%)	3 (18%)	3 (18%)	6 (35%)	6 (35%)	5 (29%)
**2**	63	34 (10)	51	15 (24%)	10 (16%)	10 (16%)	5 (8%)	42 (67%)	15 (24%)	6 (9%)
**3**	56	40 (12)	52	25 (45%)	23 (41%)	24 (44%)	5 (9%)	33 (59%)	20 (36%)	3 (5%)
